# Circulating Short-Chain Fatty Acid Levels in Chronic Kidney Disease: A Systematic Review and Meta-Analysis

**DOI:** 10.3390/nu18091440

**Published:** 2026-04-30

**Authors:** Devika Thakur, Matthew J. Harmer

**Affiliations:** 1 Department of Paediatrics, St Helier Hospital, Wrythe Ln, Carshalton SM5 1AA, UK; 2 Department of Paediatric Nephrology, Southampton Children’s Hospital, Tremona Rd, Southampton SO16 6YD, UK; matthew.harmer@uhs.nhs.uk; 3 School of Development and Health, Faculty of Medicine, University of Southampton, University Rd, Southampton SO17 1BJ, UK

**Keywords:** short chain fatty acids, dietary fibre, carbohydrates, renal function, chronic kidney disease, acetate, butyrate, propionate, gut microbiome

## Abstract

Background: Chronic kidney disease (CKD) is characterised by a disrupted gut–kidney axis, wherein intestinal dysbiosis is associated with the accumulation of uraemic toxins and the potential depletion of beneficial short-chain fatty acids (SCFAs). Whilst acetate, propionate, and butyrate are known to modulate systemic inflammation and blood pressure, their precise circulating concentrations across different CKD stages and age groups remain poorly defined. This systematic review and meta-analysis aimed to quantify blood SCFA concentrations in CKD patients compared to healthy controls. Methods: We conducted a systematic search of Medline, EMBASE, and the Cochrane Library for clinical studies reporting blood SCFA concentrations in humans with CKD. Methodological quality was assessed using the NIH tool. Standardised mean differences (SMDs) were calculated for the quantitative meta-analysis, with subgroup analyses performed for age, CKD stage, and treatment modality (dialysis vs. transplantation). Results: Twenty-one studies encompassing 9661 participants were included. Quantitative synthesis revealed a significant and consistent systemic depletion of circulating acetate and propionate in adult CKD patients compared to healthy controls (*p* < 0.05). This depletion followed a stage-dependent trajectory, worsening alongside declining glomerular filtration rates. Notably, a “butyrate paradox” was identified in paediatric cohorts; whilst adults showed progressive butyrate depletion, children with CKD often maintained or exhibited elevated levels, particularly in the context of hypertension. Furthermore, whilst haemodialysis patients exhibited the most profound SCFA deficiencies, kidney transplantation appeared to partially restore these metabolites toward healthy baseline levels. Conclusions: CKD is associated with a profound systemic reduction in acetate and propionate, supporting the model of a compromised gut–kidney axis based on converging evidence. The divergent results for butyrate in paediatric versus adult populations suggest that SCFA metabolism is influenced by age-related factors or compensatory mechanisms. These findings highlight the potential for SCFA monitoring as a candidate or emerging markers for detecting early renal damage and stratifying risk.

## 1. Introduction

SCFAs, including acetate, propionate, butyrate, and valerate, are obtained from intestinal microbial anaerobic fermentation of dietary fibre and, to a much lesser extent, directly from the diet (e.g., from vinegar, and some dairy products). Crucially, a clear distinction must be made between faecal and circulating SCFAs. While faecal SCFA measurements largely reflect the unabsorbed fraction and local colonic fermentation environment, it is the circulating SCFAs in the bloodstream that exert systemic, extra-intestinal effects. Absorption into the bloodstream primarily occurs in ionised forms via specific transporters [1]. Their myriad roles in the body include functioning as an energy source, maintaining intestinal integrity and health, regulation of carbohydrate and lipid metabolism, and regulation of inflammation [2]. SCFA status has been reported to be associated with many disease states, including metabolic, inflammatory, and oncological disease [3], with reported health benefits reported with supplementation in humans [4,5,6].

CKD represents a significant and pressing global public health issue, affecting more than 650 million people worldwide with prevalence and incidence rates rising by 40% over the past three decades, and it serves as a major multiplier for cardiovascular morbidity and mortality [7]. While traditional risk factors such as diabetes and hypertension are well-established, recent research has increasingly focused on the “gut–kidney axis,” a bidirectional communication network where gut dysbiosis acts not merely as a consequence of renal decline but as a significant driver of disease progression [8,9].

In human CKD data, clinical evidence predominantly highlights alterations in the gut microbiome, such as a marked reduction in SCFA-producing bacterial families in patients with end-stage renal disease (ESRD). Preclinical studies have suggested that the SCFA status in CKD may be mediated through the coexistent gut dysbiosis [10]. There may be a role for SCFAs to decrease inflammation, oxidative stress, and the progression of kidney disease [11,12]. Due to the altered dietary intake, altered gut microbiota, and potential link between SCFA status and cardiovascular disease [13,14], the leading cause of death in CKD [15], there is a need to explore SCFA status in those with CKD. However, human data quantifying systemic SCFA levels and are mostly limited to cohorts with advanced disease or isolated clinical parameters. Furthermore, it is essential to acknowledge age and sex as potent biological modifiers in this axis. The composition of the gut microbiome and SCFA production naturally shift with advancing age, while sex-based differences in dietary fibre intake, hormones, and metabolic rates have been shown to influence both circulating SCFA levels and the differential rate of CKD progression between men and women.

While the previous literature has broadly described gut dysbiosis in CKD, this study provides a novel contribution by systematically synthesising and quantifying the exact concentrations of circulating blood SCFAs (rather than faecal surrogates) across the entire spectrum of the disease. Herein, we synthesise the available evidence to determine whether SCFA depletion is a universal feature of CKD or if it varies by metabolite type and patient age. This systematic review and meta-analysis aims to quantify blood SCFA concentrations in CKD patients compared to healthy controls and describe reported associations.

## 2. Methods

This systematic review and meta-analysis was conducted in accordance with the Preferred Reporting Items for Systematic Reviews and Meta-Analyses (PRISMA) guidelines. The search strategy and methodology were defined by a prospective protocol, and the review was registered with PROSPERO (registration ID: CRD42024551869).

Data extraction was performed independently by two reviewers (DT and MH). Any discrepancies were resolved through discussion and consensus. The reviewers searched Medline (1946–2026), EMBASE (1974–2026), the Cochrane Library, and the reference lists of primary studies, review articles, and clinical practice guidelines on 14 January 2026. Articles were restricted to the English language. The search strategy was defined by prospective protocol. The search strategy used keywords based on the PICO question (P—subjects with chronic kidney disease (CKD); I—CKD versus no CKD; C—healthy controls; and O—short-chain fatty acid blood concentration), separated with the Boolean operator OR and combined with the Boolean operator AND. Key words were as follows: “Acetic acid” OR “Acetate” OR “Propionic acid” OR “Propionate” OR “Butyric acid” OR “Butyrate” OR “Isobutyric acid” OR “Isobutyrate” OR “Valeric acid” OR “Valerate” OR “Isovaleric acid” OR “Isovalerate” OR “2-methylbutyric acid” OR “2-Methylbutanoate” OR “Short chain fatty acids” OR “SCFA”) AND (“Chronic kidney disease” OR “CKD” OR “Chronic renal insufficiency”. Additionally, the grey literature was searched through the bibliographies of the articles included in this research. Studies were eligible for inclusion when they studied humans with CKD, were in the English language and contained data that reported the SCFA blood concentration. Studies were excluded if they were only published abstracts or were animal models or not related to the topic. Data were extracted and used to populate a synthesis table. Quality of case series was assessed using the National Heart, Lung, and Blood Institute Study Quality Assessment Tools (NIH) [16]. Data were analysed using Microsoft Excel (Microsoft Corp., Redmond, WA, USA, 2018). In addition to a narrative review of the literature, in those articles which reported blood concentrations in both CKD and in a control group, standardised mean differences were calculated to facilitate the meta-analysis with forest plot construction.

To ensure the robustness of the findings, two distinct quantitative analyses were performed for each metabolite. Analysis A (primary analysis) was restricted to studies reporting precise numerical data (mean ± SD/SEM) within the text or Supplementary Materials. Studies were excluded from the primary analysis if data required digital visual estimation from graphical plots or were mathematically reconstructed from *p*-values. Furthermore, studies reporting only relative abundances, spectral bins, or peak areas without absolute quantification were excluded from the meta-analysis to prevent data distortion but were included in the narrative synthesis. Analysis B (sensitivity analysis) was conducted to assess whether the exclusion of lower-precision data influenced the overall effect size; this inclusive analysis incorporated valid data extracted via visual estimation or statistical reconstruction. Given the anticipated clinical and methodological heterogeneity among the included observational studies, a random-effects meta-analytic model (inverse variance) was utilised. Statistical heterogeneity among the included studies was identified and quantified using the I^2^ statistic. The presence of substantial heterogeneity informed our a priori decision to conduct subgroup analyses stratifying by age (adults versus paediatrics) as well as to perform designated sensitivity analyses (Analysis B) to assess the robustness of the synthesised results. Ethical review and approval were waived for this study, as it is a systematic review and meta-analysis of previously published data.

### Results

**Figure 1:**
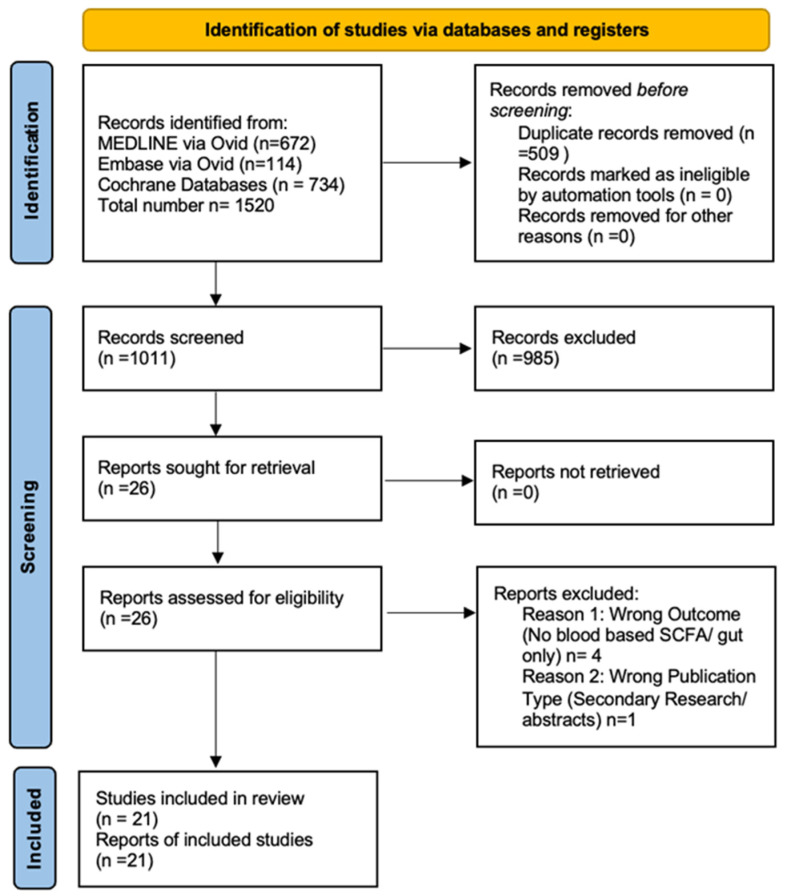
Preferred Reporting Items for Systematic Reviews and Meta- Analyses (PRISMA) flow diagram detailing the study selection process for the systematic review of short-chain fatty acid blood concentrations in chronic kidney disease.

The searches identified 1520 articles. Following screening (Figure 1), twenty-one studies were included, comprising 1938 patients in clinical cohorts and 7723 from one epidemiological analysis (total N = 9661) (Table 1).

## 3. Quality of Articles

The methodological quality of the included studies was assessed using the National Heart, Lung, and Blood Institute (NIH) Quality Assessment Tool for Case Series Studies (NIH, Bethesda, MD, USA, 2021) [16]. Of the 21 identified studies, 15 were scored as good quality (Table S1) [8,9,17,18,19,20,21,22,23,24,25,26,27,28,29]. These studies generally employed robust control groups, validated quantification methods (such as Nuclear Magnetic Resonance Spectroscopy or Gas Chromatography–Mass Spectrometry), and appropriate statistical adjustments for confounders.

The remaining studies were assessed as fair [30,31,32,33] or low quality [13,34]. Common limitations in these studies included small sample sizes, the absence of a healthy control group for baseline comparison, retrospective designs, contradictory reporting of data, or insufficient control for critical confounding variables such as dietary intake.

### 3.1. Narrative

A total of 19 clinical studies have reported SCFA blood concentrations in CKD. The cohort sizes ranged from 30 [24] to 214 [13] in clinical cohorts, with one large epidemiological analysis (NHANES) including 7723 participants [25]. Most studies were cross-sectional observational studies but did include non-randomised supplementation trials and prospective cohorts.

All major SCFAs have been reported in at least one study: acetate [9,13,17,22,34], propionate [8,9,13,26], butyrate [8,9,17,18,22,25,26], isobutyrate [8,9,17,22], valerate [9,13,21,28], and isovalerate [9,13,17].

Most studies investigated pre-dialysis CKD, although haemodialysis (HD) [8,26,35] and post-transplantation (Tx) cohorts [8] have also been examined. Of the 21 identified articles, totalling 1938 patients, acetate was the most commonly reported SCFA.

Regarding participant demographics, the sex distribution across the included cohorts was highly variable. While some studies maintained a relatively balanced male-to-female ratio [18], several cohorts were heavily skewed towards male participants, reaching up to 80% male representation in specific haemodialysis and complementary medicine cohorts [19]. The specific sex distributions for all included studies, where reported, are detailed in Table 1.

#### Do Blood Concentrations of SCFAs Differ in Those with CKD Compared to Healthy Individuals?

**Figure 2:**
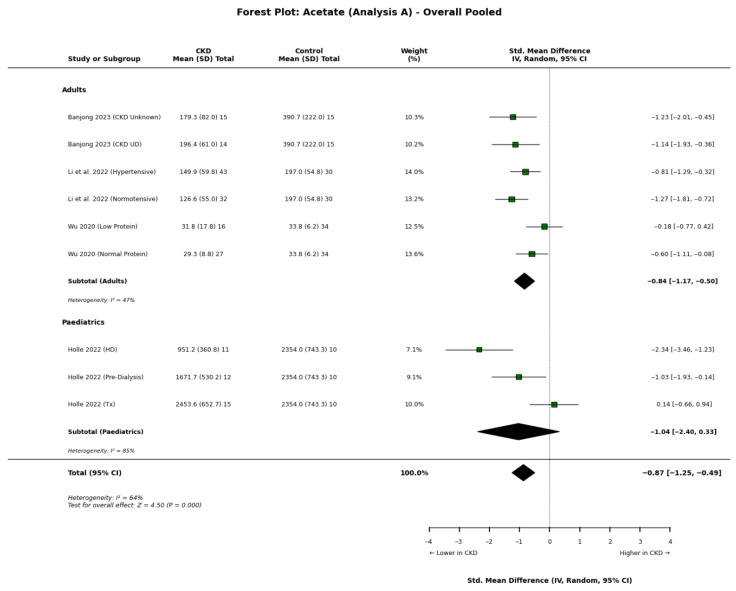
Forest plot detailing the primary meta-analysis (Analysis A) of blood acetate concentrations in patients with chronic kidney disease (CKD) compared to healthy controls, stratified by adult and paediatric populations [8,17,22,34].

Acetate: Our primary meta-analysis demonstrates a significant reduction in circulating acetate concentrations in patients with CKD compared to healthy controls. This trend was consistent across the majority of adult studies, comprising data from nine studies, totalling 868 participants (Figure 2). A stage-dependent relationship was observed between the degree of acetate depletion and the severity of renal impairment. In a large cross-sectional analysis, Jadoon et al. [13] reported plasma acetate concentrations falling from 228.6 ± 44.5 µmol/L in early-stage CKD (Stage 1–2) to 195.2 ± 29.6 µmol/L in Stage 5 (*p* < 0.01). This progressive reduction was corroborated by Fonseca et al. [27] and Wu et al. [22]. Wu et al. [9] further demonstrated that this depletion persists regardless of dietary intervention, showing reduced acetate in patients on both low-protein (31.81 μmol/L) and normal-protein diets (29.31 μmol/L) compared to controls (33.81 μmol/L). 

Metabolomic studies utilising relative quantification strongly corroborate these findings. Fonseca et al. [27] identified acetate as a key discriminator that decreased significantly as CKD advanced, while Zaki et al. [33] similarly reported that acetic acid was higher in the control group and decreased in patients with Stage 3b CKD. Contrary to these findings, Gupta et al.’s [19] Indian cohort had elevated acetate concentrations in CKD compared to controls.

**Figure 3:**
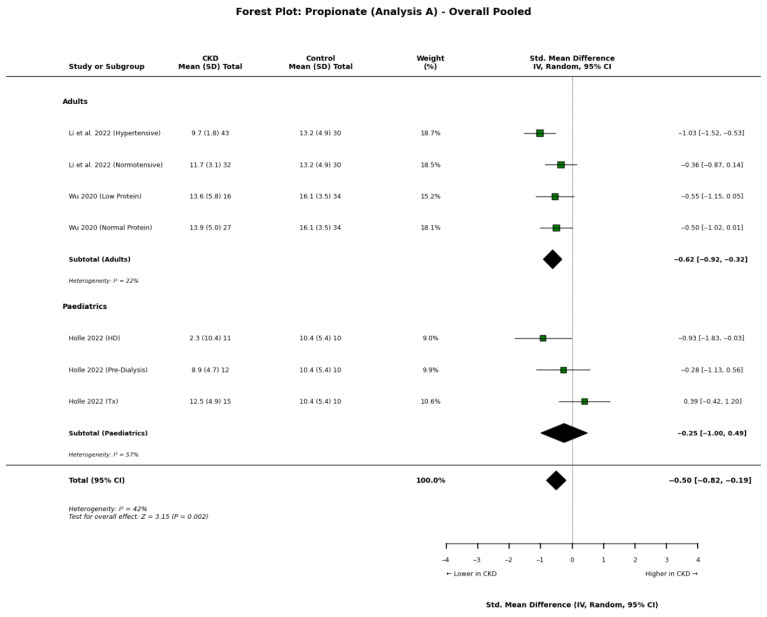
Forest plot detailing the primary meta-analysis (Analysis A) of blood propionate concentrations in patients with chronic kidney disease (CKD) compared to healthy controls, stratified by adult and paediatric populations [8,17,34].

Propionate: Similar to acetate, the meta-analysis indicates that propionate levels are significantly reduced in CKD (Figure 3). Li et al. [34] and Wu et al. [22] observed lower concentrations in adult CKD patients compared to controls. In the paediatric population, Holle et al. [8] reported starkly lower propionate, particularly in children undergoing HD (2.28 μmol/L) compared to healthy controls (10.43 μmol/L).

However, observational studies present a more heterogeneous picture. Jadoon et al. [13], in a cross-sectional analysis of 214 adults, found that while acetate decreased with advancing CKD, propionate concentrations did not differ significantly between stages 1–5, nor were they associated with prevalent cardiovascular disease. Similarly, Sokolova et al. [20] observed no significant difference in plasma propionate levels between sarcopenic and non-sarcopenic patients with CKD and heart failure, suggesting that propionate levels may be preserved in certain adult CKD subpopulations or specific comorbidities.

**Figure 4:**
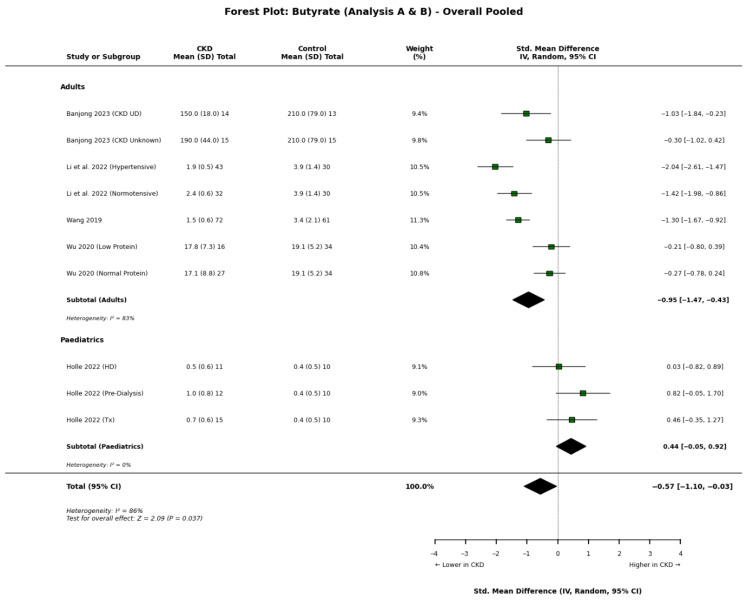
Forest plot detailing the overall pooled meta-analysis (Analyses A and B) of blood butyrate concentrations in patients with chronic kidney disease (CKD) compared to healthy controls, stratified by adult and paediatric populations [8,17,18,19,22,34].

Butyrate: The data reveal a distinct divergence between adult and paediatric populations (Figure 4). In adults, Wang et al. [18] provided the most substantial evidence for reduced butyrate in advanced disease, reporting that butyrate levels in Stage 5 CKD (1.48 μmol/L) were less than half of those in controls (3.44 μmol/L). This was supported by Li et al.’s adult CKD cohort [34]. Conversely, Holle et al. [8] found that children with pre-dialysis CKD had numerically higher butyrate levels (0.99 μmol/L) than controls (0.43 μmol/L), suggesting a possible age-dependent metabolic difference.

### 3.2. Branched-Chain SCFAs (Isobutyrate, Valerate, Isovalerate)

**Figure 5:**
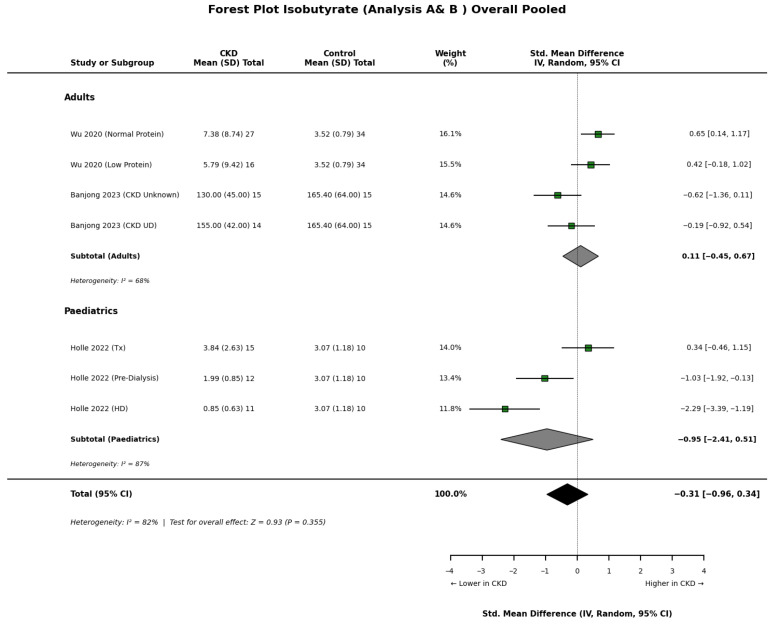
Forest plot detailing the overall pooled meta-analysis (Analyses A and B) of blood isobutyrate concentrations in patients with chronic kidney disease (CKD) compared to healthy controls, stratified by adult and paediatric populations [8,17,18,22].

**Figure 6:**
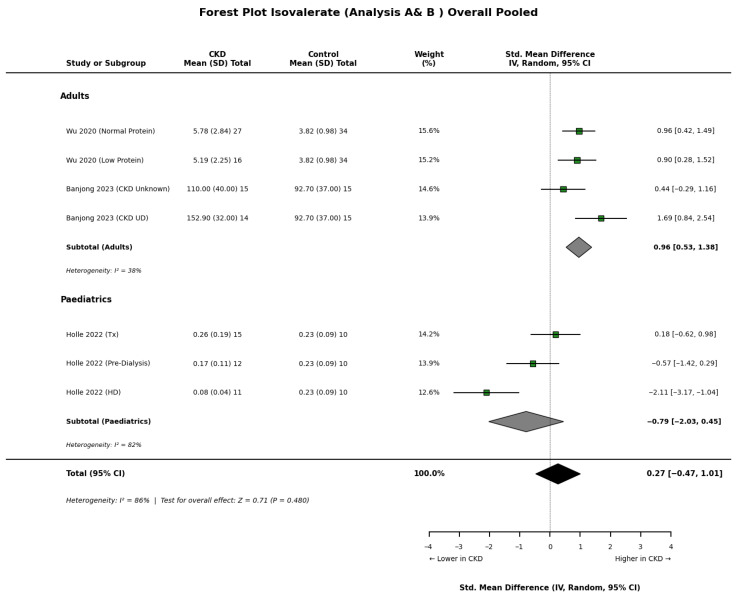
Forest plot detailing the overall pooled meta-analysis (Analyses A and B) of blood isovalerate concentrations in patients with chronic kidney disease (CKD) compared to healthy controls [8,17,18,22].

Results for branched-chain SCFAs, specifically isobutyrate (Figure 5), and isovalerate (Figure 6), demonstrated significant heterogeneity across the included cohorts. In adult populations, Wu et al. [22] reported significantly elevated serum concentrations of both isobutyrate and isovalerate in CKD patients compared to healthy controls. Conversely, paediatric data presented a different profile, with Holle et al. [8] observing significantly lower levels of isobutyrate in children with renal impairment. These age-related differences may reflect distinct metabolic shifts or dietary variations between adult and paediatric CKD populations.

**Figure 7:**
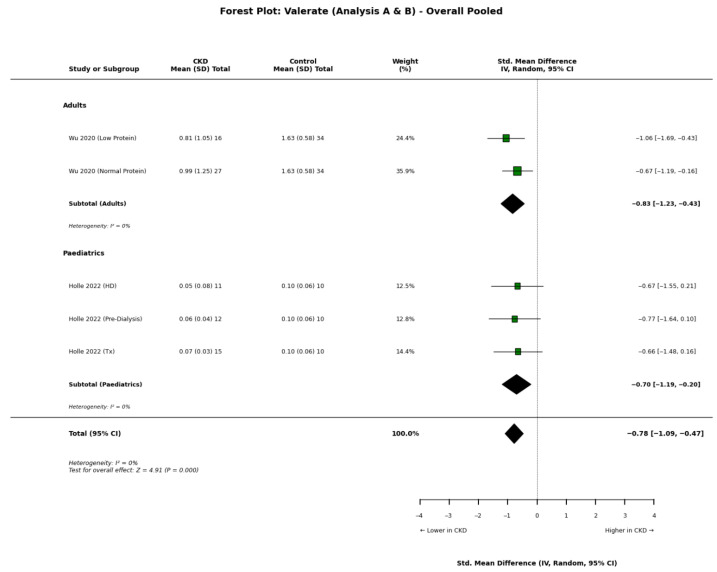
Forest plot detailing the overall pooled meta-analysis (Analyses A and B) of blood valerate concentrations in patients with chronic kidney disease (CKD) compared to healthy controls [8,22].

Findings regarding valerate concentrations were particularly discordant. Although the pooled quantitative meta-analysis indicated a general trend towards lower concentrations in CKD patients (Figure 7), individual study outcomes remained conflicting. Jadoon et al. [13] observed that valerate levels actually increased as renal disease advanced. In contrast, both Zhong et al. [21] and Wu et al. [22] reported significant systemic depletion. Notably, Zhong et al. [21] identified that this depletion served as a physiological predictor for progression to end-stage renal disease. The potential protective role of this metabolite was further supported by Mazidi et al. [28], whose Mendelian randomisation analysis suggested that higher genetically determined valerate levels are causally associated with superior renal function.

#### 3.2.1. Forest Plots as a Meta-Analysis of SCFA Blood Concentrations in CKD

The following forest plots visually detail the primary and overall pooled meta-analyses of circulating short-chain fatty acids comparing patients with CKD to healthy controls. These visualisations are stratified by adult and paediatric populations where data permits, highlighting the distinct stage- and age-dependent variations across the individual metabolites discussed in the preceding sections.

#### 3.2.2. Do Blood Concentrations of SCFAs Differ Depending upon the Degree of Renal Impairment?

Our meta-analysis indicates a graded relationship between the severity of renal impairment and circulating SCFA levels. This trend is most clearly demonstrated in the paediatric data from Holle et al. [8], with a distinct pattern across treatment modalities: concentrations of acetate and propionate were lowest in children receiving HD (acetate = 951 μmol/L), intermediate in those with pre-dialysis CKD (acetate = 1671 μmol/L), and highest in patients following kidney Tx (acetate = 2453 μmol/L). In adult non-dialysis cohorts, observational studies further support this inverse relationship between disease severity and SCFA availability. Jadoon et al. [13] documented a “progressively graded decrease” in plasma acetate concentration with advancing CKD stage, dropping from 228.6 μmol/L in Stage 1/2 to 195.2 μmol/L in Stage 5 (*p* < 0.01). This trend is supported by Wang et al.’s [18] negative correlation between butyrate levels and serum creatinine and Zhao et al. [25] reporting higher serum butyrate independent associated with preserved eGFR. Data regarding valerate remain discordant. Jadoon et al. [13] reported that plasma valerate increased with advancing CKD (*p* = 0.045). In contrast, Zhong et al. [21] found that serum valerate was significantly lower in advanced diabetic nephropathy compared to early-stage disease, and lower levels independently predicted progression to ESRD.

#### 3.2.3. Are Those in Receipt of Haemodialysis Different?

The paediatric data derived from Holle et al. [8] exhibited a distinct pattern dependent on the treatment modality. While acetate and propionate concentrations were lower in children with pre-dialysis CKD, those undergoing HD exhibited the most profound depletion (acetate: 951 μmol/L vs. 2354 μmol/L in controls; propionate: 2.28 μmol/L vs. 10.43 μmol/L). Interestingly, the higher concentrations of butyrate seen in pre-dialysis children were reversed in the HD group, returning to control levels.

Notably, patients who had undergone kidney Tx displayed SCFA concentrations comparable to, or higher than, healthy controls. Crucially, this metabolic restoration tracked with renal function; the transplant cohort had a mean eGFR of 78.8 ± 19.4 mL/min/1.73 m^2^, significantly higher than both the pre-dialysis (mean eGFR 29.6) and HD groups (mean eGFR 6.6). 

#### 3.2.4. In Those with CKD, How Does Diet Influence Blood Concentrations of SCFA?

Wu et al. [9] investigated the impact of a low-protein diet (LPD) on SCFA levels. Patients receiving LPD (0.6−0.8 g/kg/day) exhibited significantly lower serum levels of acetic acid, heptanoic acid, and nonanoic acid compared to those on a normal protein diet and healthy controls. This reduction was linked to a significant decrease in the abundance of butyrate-producing bacteria (families Lachnospiraceae and Bacteroidaceae) [9]. Additionally, Zhao et al. [25] analysed NHANES data and found that higher dietary butyrate intake was independently associated with higher eGFR, suggesting that dietary consumption directly influences circulating levels and renal outcomes.

#### 3.2.5. Does the Aetiology of CKD Influence Blood Concentrations of SCFA?

Hsu et al. [30] compared children with congenital anomalies of the kidney and urinary tract (CAKUT) against non-CAKUT CKD. They found that children with CAKUT had significantly lower plasma levels of propionate compared to the non-CAKUT group, although acetate and butyrate levels did not differ. In adults, Zhong et al. [21] focused specifically on diabetic nephropathy (DN), finding that valerate and caproate levels were lower in advanced DN compared to early DN. Banjong et al. [17] noted that patients with CKD of unknown aetiology (CKDu) had SCFA profiles similar to those with defined underlying diseases, though both were lower than healthy controls.

#### 3.2.6. In Those with CKD, Is There an Association Between SCFA Blood Concentrations and Blood Pressure and/or Associated Cardiovascular Measures?

Li et al. [34] observed that while acetate, butyrate, and propionate were lower than healthy controls in all CKD groups, levels were numerically different between hypertensive and normotensive CKD patients. Paradoxically, Hsu et al. [30] reported that in children with CKD, higher plasma propionate levels were positively correlated with systolic blood pressure (r = 0.288) and abnormal ambulatory blood pressure profiles. Lu et al. [32] found that in a longitudinal analysis, higher index plasma butyrate and propionate levels were associated with a higher probability of worsened blood pressure and decreased ejection fraction over one year. Conversely, Jadoon et al. [13] found that higher valerate levels were independently associated with prevalent cardiovascular disease and coronary artery disease in adults. However, Gupta et al. [19] noted that decreased lipid and membrane metabolites in CKD resembled profiles seen in acute myocardial infarction, linking metabolic depletion to cardiovascular risk.

**Figure 8:**
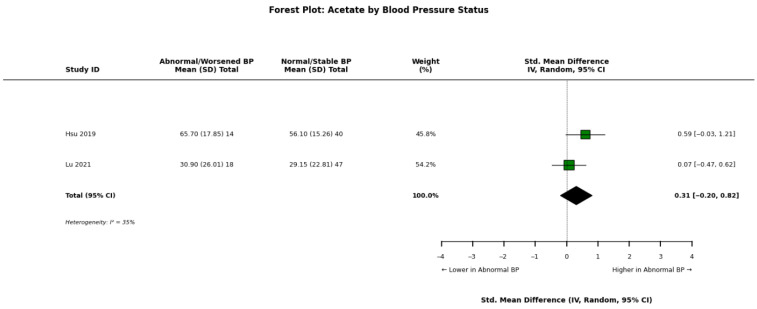
Forest plot detailing the meta-analysis of blood acetate concentrations in patients with chronic kidney disease (CKD), stratified by abnormal/worsened versus normal/stable blood pressure status [30,32].

**Figure 9:**
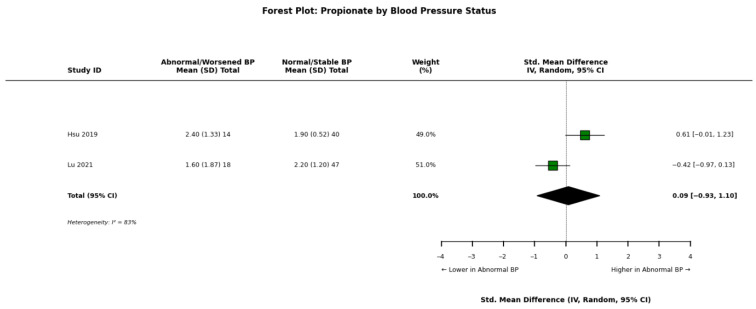
Forest plot detailing the meta-analysis of blood propionate concentrations in patients with chronic kidney disease (CKD), stratified by abnormal/worsened versus normal/stable blood pressure status [30,32].

**Figure 10:**
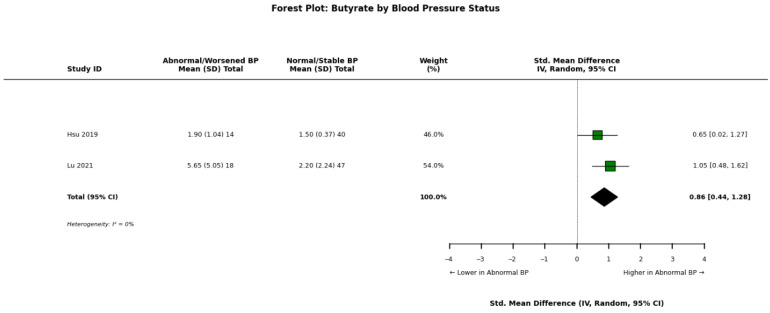
Forest plot detailing the meta-analysis of blood butyrate concentrations in patients with chronic kidney disease (CKD), stratified by abnormal/worsened versus normal/stable blood pressure status [30,32].

A pooled meta-analysis of these findings across abnormal/worsened versus normal/stable blood pressure groups is visually detailed for acetate (Figure 8), propionate (Figure 9), and butyrate (Figure 10).

#### 3.2.7. Does Supplementation of SCFA Improve Outcomes in Those with CKD?

Interventional data suggests potential benefits. Marzocco et al. [35] demonstrated that oral propionate supplementation in HD patients led to a reduction in systemic inflammation (IL-2, IL-17) and a non-significant trend toward lower systolic blood pressure. In a separate intervention, Li et al. [26] showed that supplementation with a soluble dietary fibre mixture for eight weeks in HD patients significantly increased serum butyric acid, isobutyric acid, and valeric acid levels compared to placebo, which correlated with improved haemoglobin and iron metabolism indices. Furthermore, Zhao et al. [24] found that treatment with roxadustat (an HIF-PHI) increased serum SCFA levels and the abundance of SCFA-producing bacteria, which coincided with alleviated inflammatory status. However, it is critical to emphasise that SCFA supplementation or direct microbiome-targeted therapies cannot currently be recommended in routine clinical practice. The existing interventional trials are small, short-term, and largely limited to haemodialysis cohorts. Furthermore, the successful outcomes reported thus far are predominantly restricted to surrogate biochemical or inflammatory markers rather than hard clinical endpoints (such as cardiovascular events or mortality). Therefore, while these preliminary findings are hypothesis-generating, such interventions should remain strictly within the realm of clinical trials until robust, long-term data validate their clinical efficacy and safety.

#### 3.2.8. Q. Are Findings Different for Children and Young People Compared to Adult CKD Patients?

A distinct age-related divergence was observed for butyrate. While multiple adult studies [17,18,34] reported a depletion of serum butyrate in CKD, the paediatric data from Holle et al. [8] showed an accumulation of butyrate in pre-dialysis children compared to controls. However, for acetate and propionate, both paediatric and adult cohorts showed consistent depletion in the disease state. Additionally, the association between SCFAs and blood pressure appears more paradoxical in children, where higher concentrations of propionate/butyrate were linked to worse BP parameters, whereas in adults, SCFAs are generally considered vasodilatory and protective.

## 4. Discussion

In this systematic review and meta-analysis, we synthesised data from 21 studies to evaluate the blood concentrations of SCFAs in patients with CKD. This is the first review to explicitly contrast adult and paediatric data while incorporating recent evidence regarding the influence of hypertension, diabetic kidney disease, and pharmacological interventions. Our quantitative meta-analysis supports the ‘SCFA deficiency hypothesis,’ confirming a consistent systemic depletion of acetate and propionate in the adult CKD population, which is consistent with a model in which SCFA depletion accompanies CKD progression. However, our findings also reveal critical nuances regarding disease stage, treatment modality, and age that challenge a ‘one-size-fits-all’ interpretation of gut–kidney axis dysbiosis.

The observed universal depletion of acetate and propionate may reflect the hostile uraemic environment, which selectively suppresses saccharolytic bacterial families such as *Lachnospiraceae* and *Ruminococcaceae* [18,22]. Consequently, circulating SCFA concentrations generally exhibit a graded inverse relationship with CKD severity, decreasing progressively as renal function declines [13]. However, it is important to note that these circulating concentrations represent a complex net balance of microbial production, intestinal absorption, host tissue utilisation, and renal clearance [2]. With this metabolic balance in mind, this progressive depletion supports the model of a bidirectional ‘gut–kidney axis’: declining renal function correlates with the accumulation of uraemic toxins in the gut lumen, which may raise luminal pH and favour proteolytic over saccharolytic fermentation [18]. Consequently, the production of potentially protective SCFAs drops, a shift that is hypothesised to further compromise the gut barrier and associate with accelerated renal damage. Notably, branched-chain SCFAs (BC-SCFAs) and related metabolites such as valerate do not consistently follow this linear decline. Unlike acetate and propionate, which are primarily derived from saccharolytic fermentation, BC-SCFAs are predominantly the products of protein fermentation. Therefore, their circulating concentrations may reflect distinct metabolic axes, representing a complex interplay of dietary protein load, muscle catabolism, and/or a shift towards proteolytic dysbiosis in the uraemic gut. While it is plausible that the persistence or elevation of valerate in advanced end-stage renal disease serves as a marker for muscle breakdown and high protein turnover, this interpretation remains speculative and has not yet been directly demonstrated in clinical cohorts [13,20].

The substantial clinical and methodological heterogeneity among the included studies must be explicitly acknowledged, as it heavily informs the interpretation of our results and contextualises several discordant findings. First, disagreeing findings regarding specific metabolites can be largely attributed to variations in underlying disease aetiology and comorbidities. For example, while Jadoon et al. reported that circulating valerate increased with advancing CKD and was positively associated with cardiovascular disease [13], Zhong et al. conversely found that valerate was significantly depleted in advanced diabetic nephropathy and that lower levels predicted progression to end-stage renal disease [21]. Similarly, Sokolova et al. identified specific SCFA alterations, notably hexanoic acid, that were uniquely associated with sarcopenia in patients with concomitant CKD and heart failure [20]. Furthermore, Hsu et al. demonstrated that paediatric patients with congenital anomalies of the kidney and urinary tract (CAKUT) exhibited significantly lower propionate compared to those with acquired non-CAKUT diseases [30]. This suggests that the inflammatory milieu and specific comorbidities of distinct CKD subpopulations uniquely shape the microbial metabolic output.

Second, variations in diet, ethnicity, and geography play a profound role in determining microbiome composition and SCFA generation. The studies included in this review span vastly diverse populations, including cohorts from China, Taiwan, Thailand, India, Germany, Italy, Brazil, Mexico, and the United States. Differences in regional dietary habits, cultural fibre intake, and environmental exposures drastically alter substrate availability; for instance, Banjong et al. noted that their rural Thai cohort’s agricultural lifestyle and potential pesticide exposure could uniquely impact the microbiome in CKD of unknown aetiology (CKDu) [17]. Gupta et al. also highlighted geographical and cultural heterogeneity by demonstrating distinct metabolic profiles in Indian CKD patients utilising complementary and alternative medicine (CAM) [19]. Furthermore, specific clinical dietary interventions alter these profiles; strict low-protein diets prescribed to CKD patients independently contract butyrate-producing bacterial populations and significantly reduce serum SCFAs [22].

Finally, methodological heterogeneity in analytical techniques contributes to conflicting results. The studies in this review utilised diverse quantification platforms, including Gas Chromatography–Mass Spectrometry (GC-MS) [13,21] Liquid Chromatography–Mass Spectrometry (LC-MS/MS), Nuclear Magnetic Resonance (1H-NMR) spectroscopy [19,27,29], and GC–Flame Ionisation Detection (GC-FID) [32]. The differences in sensitivity between these platforms, the variance between absolute quantification versus relative abundance reporting (such as NMR spectral bins) [27], and the lack of standardisation in sample preparation protocols (e.g., vacuum distillation versus solvent extraction) underscore why some studies detect significant alterations while others do not.

A distinct age-dependent divergence exists regarding butyrate. While adult cohorts exhibit progressive depletion associated with adverse outcomes, paediatric populations display a ‘butyrate paradox,’ characterised by preserved or elevated circulating levels [8]. However, these observations rely heavily on a highly limited paediatric evidence base. Consequently, any mechanistic explanations for this divergence currently remain speculative. It is hypothesised that the paediatric microbiome might possess a plasticity or resilience lost in the aged, comorbid adult gut, or alternatively, this may simply reflect unmeasured dietary disparities. Healthy control children in Western cohorts often consume ‘typical’ diets high in processed foods and lower in fibre, whereas children with CKD receive rigorous dietetic counselling. Consequently, despite potassium restrictions, these children may consume higher-quality diets than their ‘healthy’ peers. To definitively determine whether this ‘butyrate paradox’ is driven by true biological resilience or merely environmental and methodological confounders, there is an urgent need for diet-controlled, age-specific longitudinal studies.

Treatment modality further influences this metabolic profile. Patients undergoing haemodialysis exhibit the most profound depletion of acetate and propionate [8]. In addition to stringent dietary restrictions and the clearance of water-soluble SCFAs into the dialysate, the physiological stress of the procedure itself warrants consideration. Haemodynamic instability during dialysis is associated with splanchnic hypoperfusion, which may induce transient intestinal ischaemia and compromise epithelial barrier integrity [36,37]. Conversely, kidney transplantation appears to successfully restore the metabolic profile to near-healthy baselines, suggesting that the restoration of glomerular filtration and the subsequent clearance of the uraemic milieu are critical for the recovery of SCFA metabolism [8].

There is a clear therapeutic tension between traditional renoprotection and microbiome maintenance. Dietary protein restriction exacerbates SCFA depletion by contracting butyrate-producing guilds, likely because patients reduce overall plant-food intake to control protein and potassium loads [9]. It is important to note that current interventional findings, such as dietary fibre or propionate supplementation, must be considered preliminary and hypothesis-generating, with reported outcomes largely limited to surrogate biomarkers rather than hard clinical endpoints. Nevertheless, the microbiome appears responsive; preliminary trials with soluble fibre or high-amylose starch have successfully augmented circulating SCFAs [26]. Furthermore, pharmacological interventions targeting non-microbial pathways appear to exert beneficial ‘off-target’ effects. For example, the anaemia treatment roxadustat and the uraemic-toxin-adsorbent AST-120 have both been shown to increase serum SCFA levels, implying that managing the uraemic environment promotes microbial recovery independent of dietary intake [24,31]. SCFA supplementation or direct microbiome-targeted therapies cannot currently be recommended in routine clinical practice. The existing interventional trials are small, short-term, and largely limited to haemodialysis cohorts. Furthermore, the successful outcomes reported thus far are predominantly restricted to surrogate biochemical or inflammatory markers rather than hard clinical endpoints (such as cardiovascular events or mortality). Therefore, while these preliminary findings are hypothesis-generating, such interventions should remain strictly within the realm of clinical trials until robust, long-term data validate their clinical efficacy and safety.

Beyond disease stage and diet, the aetiology of CKD imprints a unique metabolic signature. Metabolic aetiologies such as diabetic nephropathy are associated with specific depletions in valerate, likely linked to insulin resistance and altered lipid metabolism [21]. In children, those with CAKUT exhibit significantly lower propionate levels compared to those with glomerular diseases [30]. This suggests that the inflammatory milieu or immunological treatments associated with acquired glomerular disease may select for distinct microbial guilds (such as the propionate-producing *Phascolarctobacterium*) that are absent in the non-inflammatory structural defects of CAKUT.

The physiological consequences of these metabolic shifts are most evident in cardiovascular regulation. In adults, low SCFAs correlate with hypertension and vascular stiffness, consistent with the vasodilatory role of GPR41/43 activation [13]. Paradoxically, in children, elevated propionate correlates with higher blood pressure [30]. This contradiction may reflect the differential activation of Olfactory Receptor 78 (Olfr78), which raises blood pressure via renin release These cardiovascular signalling mechanisms—including the differential roles of GPR41, GPR43, and Olfr78—are currently extrapolated from the experimental animal literature [38]. While it is hypothesised that the pressor Olfr78 pathway may dominate in the developing paediatric kidney, whereas the loss of vasodilatory GPR41/43 signalling drives pathology in the rigid vasculature of adult CKD, this interpretation remains speculative. Rigorous human validation is urgently required to confirm these specific receptor pathways, particularly within paediatric CKD cohorts where clinical translational data remain exceptionally sparse.

Immunologically, SCFA depletion aligns with the ‘inflammaging’ hypothesis, where the loss of microbial metabolites releases the immunological ‘brake’ on pro-inflammatory cytokines such as TNF-α and IL-6 [8,34]. Emerging evidence suggests that enhancing SCFA availability, whether through soluble fibre or direct supplementation, can mitigate this inflammation and potentially preserve glomerular filtration [34,35]. However, we must be cautious in interpreting low SCFA levels as purely pathological. It is essential to consider that low systemic concentrations might, in some contexts, represent a protective host adaptation rather than a purely pathological deficit. Therefore, while ‘microbiome-sparing’ therapies hold promise, therapeutic supplementation must be approached with caution until causal evidence is established.

Our review has limitations. The high heterogeneity in meta-analyses reflects variations in quantification methods (NMR vs. Mass Spectrometry) and biological variability. Most included studies were cross-sectional, preventing causal inference. Additionally, few studies controlled for dietary intake, which is the major determinant of SCFA production. Furthermore, a crucial limitation is that reduced systemic circulating SCFAs cannot be exclusively interpreted as diminished microbial production in the gut. Rather, circulating blood levels reflect a complex, dynamic balance between intestinal microbial generation, colonic mucosal absorption, subsequent hepatic and peripheral host utilisation, and renal clearance. For instance, the liver clears a major fraction of portal propionate and butyrate, while acetate is extensively utilised by peripheral tissues; therefore, altered blood SCFA profiles in CKD may partially reflect shifts in host metabolism, altered transporter expression, or reduced renal clearance rather than dysbiosis alone. Furthermore, the fasting status of participants and the specific timing of blood collection were not universally standardised or consistently reported across all included studies. As circulating SCFA concentrations can fluctuate post-prandially and diurnally, this introduces a potential source of methodological heterogeneity and remains a limitation of the current evidence base.

Another major limitation of the current evidence base—and consequently this meta-analysis—is the widespread failure of primary studies to address sex as a biological variable. Sex exerts a profound influence on both SCFA metabolism and CKD progression. Epidemiological evidence suggests that men frequently exhibit lower circulating SCFA levels compared to women, a discrepancy that may be partially driven by historically lower general dietary fibre intake among males. Furthermore, men often progress to end-stage renal disease at a faster rate and exhibit higher blood pressure and cardiovascular risk profiles than premenopausal women, whose elevated oestrogen concentrations are hypothesised to offer renoprotection against oxidative multi-organ damage. Because the majority of the included studies did not stratify their SCFA quantification by sex and several cohorts were disproportionately male, the true impact of sex-specific hormonal and dietary differences on the gut–kidney axis remains obscured. This limits the generalisability of the pooled findings and underscores a critical gap in the literature. Finally, the majority of the data is derived from adult populations, limiting the strength of conclusions for paediatric CKD. Future clinical practice should consider serum acetate and valerate as potential non-invasive biomarkers for detecting early renal damage, but therapeutic strategies must be tailored to the patient’s age and disease stage.

In this systematic review and meta-analysis, we synthesised data from 21 studies to evaluate the blood concentrations of SCFAs in patients with CKD. This is the first review to explicitly contrast adult and paediatric data while incorporating recent evidence regarding the influence of hypertension, diabetic kidney disease, and pharmacological interventions. Our quantitative meta-analysis is consistent with a model in which SCFA depletion may accompany CKD progression (specifically acetate and propionate). However, our findings also reveal critical nuances regarding disease stage, treatment modality, and age that challenge a ‘one-size-fits-all’ interpretation of gut–kidney axis dysbiosis.

Future Directions: While the current evidence robustly links SCFA depletion to CKD progression, translating these findings into clinical practice requires several key steps. First, circulating SCFAs, particularly serum acetate and valerate, must be viewed as emerging candidate markers requiring proper validation rather than established diagnostic tools. Their true diagnostic and prognostic utility must be rigorously validated in large, prospective, diet-controlled longitudinal human cohorts. Furthermore, age-specific studies are urgently needed to definitively elucidate the divergent metabolic profiles observed between paediatric and adult populations. Finally, future clinical trials must evaluate whether targeted ‘microbiome-sparing’ therapies—such as specific soluble fibre supplementation, the oral adsorbent AST-120, or hypoxia-inducible factor prolyl hydroxylase inhibitors (e.g., roxadustat)—can successfully restore metabolic and immune homeostasis, translate into hard clinical endpoints, and safely delay disease progression across different patient demographics.

## 5. Conclusions

This systematic review and meta-analysis synthesises available evidence to demonstrate that CKD is characterised by a significant systemic depletion of key SCFAs, specifically acetate and propionate, which correlates with the decline in glomerular filtration rate. These findings support the model of a disrupted gut–kidney axis, where uraemia-associated dysbiosis is linked to a loss of metabolic signalling that may be essential for maintaining immune homeostasis and vascular health.

Crucially, our analysis uncovers a divergent age-dependent profile regarding butyrate. While adult cohorts exhibit progressive depletion associated with adverse renal and cardiovascular outcomes, paediatric populations display a ‘butyrate paradox’ characterised by preserved or elevated circulating levels that may hold distinct pathophysiological associations with blood pressure. This discrepancy challenges the universality of current dysbiosis models and suggests that adult-derived therapeutic strategies cannot be automatically extrapolated to children.

We further identify that SCFA status is modifiable. Whereas HD and standard protein-restricted diets may inadvertently exacerbate SCFA depletion, specific interventions, including soluble fibre supplementation, the oral adsorbent AST-120, and the hypoxia-inducible factor prolyl hydroxylase inhibitor roxadustat can successfully augment circulating SCFA levels. Notably, kidney Tx appears to be the most effective intervention for restoring the metabolic profile to near-healthy baselines.

Future clinical practice should consider serum acetate and valerate as potential non-invasive biomarkers for detecting early renal damage and stratifying risk. Ultimately, therapeutic strategies must be tailored to the patient’s age and disease stage, moving towards ‘microbiome-sparing’ renal care that prioritises the maintenance of the gut fermentative capacity to delay disease progression.

## 6. Concluding Remarks

CKD is characterised by systemic SCFA depletion, with distinct age- and treatment-related patterns. SCFAs may serve as biomarkers for renal progression and cardiovascular risk, and targeted interventions could restore metabolic and immune homeostasis.

## Figures and Tables

**Table 1:** Summary of characteristics and key findings of the studies included in the systematic review (*n* = 21).

Author and Year	Population (Age)	Study-Design	Key- Findings	Limitations	Quality Rating
Holle et al. (2022) [8]	Paediatric (3–18 years, 56% male)	Cross-sectional	Acetate and propionate depleted in haemodialysis; restored post-transplant. Depletion linked to inflammation.	Small sample size; cross-sectional design; cardiovascular phenotype limited to office BP.	Good
Banjong et al. (2023) [17]	Adults (~63 years, 39% male	Cross-sectional	Serum acetate significantly lower in early-stage CKD. Lowest SCFA levels found in those with underlying disease.	Small sample size (*n* = 72); parasitic infection confounder (screened); diet not strictly controlled.	Good
Wang et al. (2019) [18]	Adults (46–52 years, 50% male	Cross-sectional + animal	Butyrate significantly reduced in CKD 5. Lower butyrate correlated with worse renal function.	Single centre; included autoimmune comorbidities; cross-sectional human data.	Good
Gupta et al. (2023) [19]	Adults (43–49 years, 83% male)	Cross-sectional	Acetate identified as key discriminator (AUC > 0.9); CAM users showed profiles closer to controls.	Relative quantification (NMR bins); small sample size; lifestyle confounders.	Good
Sokolova et al. (2026) [20]	Elderly (~70 years, 53% male)	Cross-sectional	Hexanoic acid (C6) independently associated with sarcopenia (OR 2.24) and inflammation.	No healthy control group (all had heart failure/CKD); cross-sectional.	Good
Zhong et al. (2022) [21]	Adults (52 ± 9 years, 71% male)	Retrospective cohort	Valerate and caproate significantly lower in advanced diabetic nephropathy (DN).	No healthy control group; retrospective design; single centre.	Good
Wu et al. (2020a) (Nutrients) [22]	Elderly (63–66 years, 49% male)	Cross-sectional (intervention)	Low-protein diet associated with lower serum acetate and heptanoic and nonanoic acid.	Small sample size; short dietary instruction (3 mos); observational adherence.	Good
Wu et al. (2020b) (Theranostics) [9]	Adults (64 ± 7 years, 48% male)	Cross-sectional	Propionate decreased in advanced CKD. Severity-specific microbial signatures identified.	Cross-sectional design prevents causal inference.	Good
Wu et al. (2024) (NDT) [23]	Adults (63 ± 5 years, 48% male)	Cross-sectional	Valeric and hexanoic acid levels lower in CKD. Linked to B-cell immunity.	Cross-sectional design; single centre.	Good
Zhao et al. (2023) [24]	Adults (54 ± 17 years, 57% male)	Prospective self-controlled	Roxadustat treatment increased serum SCFA levels and SCFA-producing bacteria.	No healthy control group; small sample size; lack of placebo arm.	Good
Zhao et al. (2025) [25]	Adults (59–61 years, 63% male)	Dual-cohort observational	Higher dietary and serum butyrate associated with preserved eGFR.	Dietary recall bias (NHANES); clinical cohort small (*n* = 70) and lacked healthy controls.	Good
Li et al. (2022b) (J Transl Med) [26]	Adults (50 ± 8 years, 54% male)	RCT	Soluble fibre increased serum butyrate, valerate, and hexanoic acid and improved renal anaemia.	Single centre; short intervention (8 weeks); specific fibre type.	Good
Fonseca et al. (2023) [27]	Adults (sSex NR)	Cross-sectional	Acetate is identified as a key discriminator decreasing with CKD stage (lowest in G5).	Small control group; relative quantification (NMR bins).	Good
Mazidi et al. (2023) [28]	Adults (sex NA)	Mendelian randomisation	Genetically determined higher plasma valerate causally associated with better renal function.	Relies on summary statistics; limited genetic instruments for other SCFAs.	Good
Lucio-Gutiérrez et al. (2024) [29]	Adults (~52% male)	Cross-sectional	Acetate-to-creatinine ratio identified as biomarker for early renal damage in T2D.	Small sample size; specific to diabetic population; relative quantification.	Good
Hsu et al. (2019) [30]	Children (11.2 years, 63% male)	Cross-sectional	Propionate lower in CAKUT vs. non-CAKUT. High propionate linked to abnormal BP (paradoxical).	No healthy control group; single centre; diet not controlled.	Fair
Hsu et al. (2022) [31]	Adults (66 ± 7 years, 43% male)	Interventional	AST-120 treatment increased serum acetate and octanoic acid.	Very small sample in treatment group (*n* = 8); non-randomized selection.	Fair
Lu et al. (2021) [32]	Children (9.6 years, 57% male)	Longitudinal cohort	Acetate increased in stable BP group; butyrate increased in worsened BP group.	No healthy control group; small subgroup sizes; retrospective analysis.	Fair
Zaki et al. (2025) [33]	Adults (66 ± 1 years, 70% male)	Case–control	Acetic acid and indole propionic acid lower in CKD. Butyrate reported as increased.	Contradictory reporting on butyrate direction; relative quantification; no diet data.	Fair
Jadoon et al. (2018) [13]	Adults (55–68 years, 51% male)	Cross-sectional	Valerate higher in CVD patients. Acetate decreased with advancing CKD stage.	No healthy control group (internal comparison only); lack of dietary data.	Low
Li et al. (2022a) (Circ Res) [34]	Adults (sex NR)	Cross-sectional + animal	Butyrate and acetate reduced in CKD. F. prausnitzii protects kidney via butyrate-GPR43 axis.	Human data reporting limited (inconsistencies in patient numbers); primary focus on mouse model.	Low

## Data Availability

No new data were created or analysed in this study. Data sharing is not applicable to this article.

## Supplementary Materials

The following supporting information can be downloaded at: https://www.mdpi.com/article/10.3390/nu18091440/s1, Table S1: Methodological quality assessment of the included studies using the NIH Quality Assessment Tool; Figure S1: Sensitivity analysis for the meta-analysis of blood acetate concentrations in CKD patients versus healthy controls; Figure S2: Sensitivity analysis for the meta-analysis of blood propionate concentrations in CKD patients versus healthy controls.
