# Supplementary material for: Circulating Short-Chain Fatty Acid Levels in Chronic Kidney Disease: A Systematic Review and Meta-Analysis
Source: Nutrients. 2026 Apr 30;18(9):1440. doi: 10.3390/nu18091440 (PMC13164688; doi:10.3390/nu18091440)
Supplement: Supplementary file 1 [file nutrients-18-01440-s001.zip › nutrients-4213217-supplementary.pdf]

Table S1: Methodological quality assessment of the included studies using the NIH Quality Assessment Tool (Case Series Studies)

Study (Author & Year)	Q1	Q2	Q3	Q4	Q5	Q6	Q7	Q8	Q9	Overall Quality Rating
Holle et al. (2022)	Y	Y	Y	Y	NA	Y	NA	Y	Y	Good
Banjong et al. (2023)	Y	Y	Y	Y	NA	Y	NA	Y	Y	Good
Wang et al. (2019)	Y	Y	Y	Y	NA	Y	NA	Y	Y	Good
Gupta et al. (2023)	Y	Y	Y	Y	NA	Y	NA	Y	Y	Good
Sokolova et al. (2026)	Y	Y	Y	N	NA	Y	NA	Y	Y	Good
Zhong et al. (2022)	Y	Y	Y	N	NA	Y	Y	Y	Y	Good
Wu et al. (2020a)	Y	Y	Y	Y	Y	Y	NA	Y	Y	Good
Wu et al. (2020b)	Y	Y	Y	Y	NA	Y	NA	Y	Y	Good
Wu et al. (2024)	Y	Y	Y	Y	NA	Y	NA	Y	Y	Good
Zhao et al. (2023)	Y	Y	Y	N	Y	Y	Y	Y	Y	Good

Zhao et al. (2025)	Y	Y	Y	N	NA	Y	Y	Y	Y	Good
Li et al. (2022b)	Y	Y	Y	Y	Y	Y	Y	Y	Y	Good
Fonseca et al. (2023)	Y	Y	Y	Y	NA	Y	NA	Y	Y	Good
Mazidi et al. (2023)	Y	Y	Y	Y	NA	Y	NA	Y	Y	Good
Lucio-Gutiérrez et al. (2024)	Y	Y	Y	Y	NA	Y	NA	Y	Y	Good
Hsu et al. (2019)	Y	Y	Y	N	NA	Y	NA	Y	Y	Fair
Hsu et al. (2022)	Y	Y	CD	N	Y	Y	Y	Y	Y	Fair
Lu et al. (2021)	Y	Y	Y	N	NA	Y	Y	Y	Y	Fair
Zaki et al. (2025)	Y	Y	Y	Y	NA	CD	NA	Y	N	Fair
Jadoon et al. (2018)	Y	Y	CD	N	NA	Y	NA	Y	CD	Low
Li et al. (2022a)	Y	N	CD	CD	NA	N	NA	Y	N	Low

Table Key: Y = Yes; **N** = No; **CD** = Cannot Determine; **NA** = Not Applicable; **NR** = Not Reported.

NIH Quality Assessment Tool for Case Series Studies Questions:

- Q1: Was the study question or objective clearly stated?
- Q2: Was the study population clearly and fully described, including a case definition? *(Scored 'No' for studies with inconsistent reporting of human patient numbers)*.
- Q3: Were the cases consecutive?
- Q4: Were the subjects comparable? *(Scored 'No' for studies lacking a healthy control group for baseline comparison)*.
- Q5: Was the intervention clearly described? *(Scored 'NA' for cross-sectional observational studies without a specific clinical intervention)*.
- Q6: Were the outcome measures clearly defined, valid, reliable, and implemented consistently across all study participants?
- Q7: Was the length of follow-up adequate? *(Scored 'NA' for single time-point, cross-sectional studies)*.
- Q8: Were the statistical methods well-described?
- Q9: Were the results well-described? *(Scored 'No' or 'CD' for studies with contradictory reporting of key outcomes, such as butyrate directionality)*.

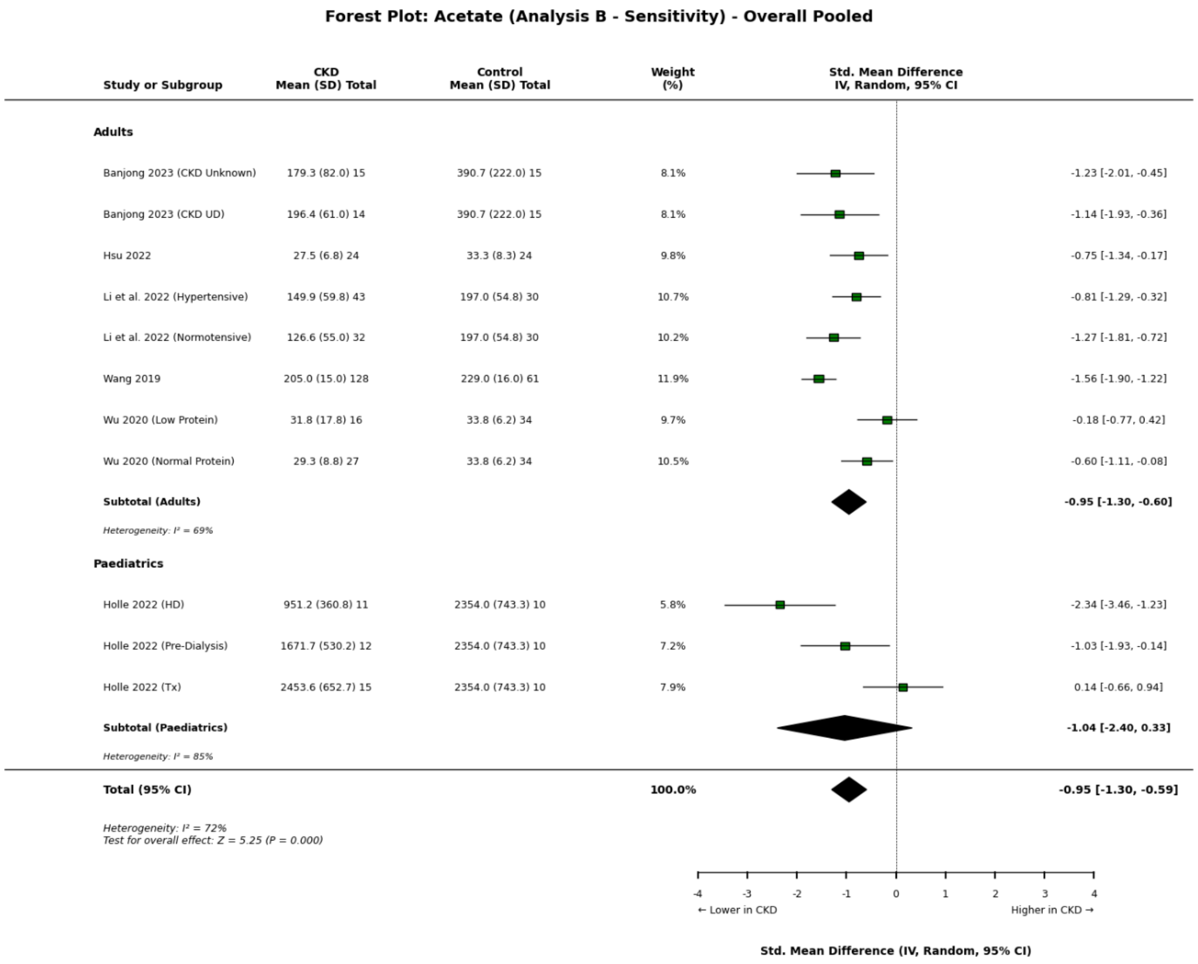

Figure S1. *Sensitivity analysis for the meta-analysis of blood acetate concentrations in CKD patients vs. healthy controls.*

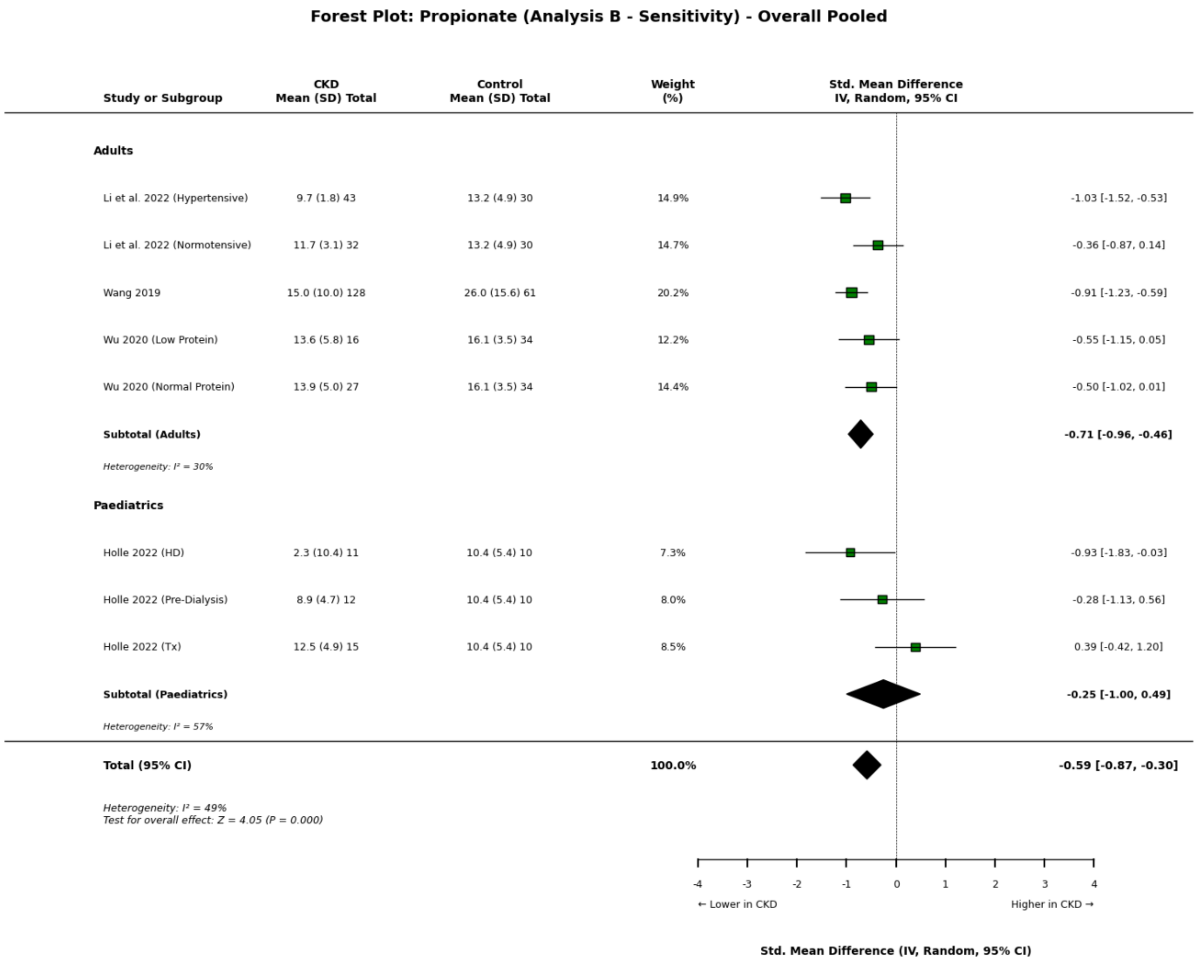

Figure S2. *Sensitivity analysis for the meta-analysis of blood propionate concentrations in CKD patients vs. healthy controls.*
